# Can Physical Activity Reduce the Risk of Cognitive Decline in Apolipoprotein e4 Carriers? A Systematic Review

**DOI:** 10.3390/ijerph18147238

**Published:** 2021-07-06

**Authors:** Jose Luis Perez-Lasierra, Jose Antonio Casajús, José Antonio Casasnovas, Jose Miguel Arbones-Mainar, Antonio Lobo, Elena Lobo, Belén Moreno-Franco, Alejandro Gonzalez-Agüero

**Affiliations:** 1 Department of Physiatry and Nursing, Universidad de Zaragoza, 50009 Zaragoza, Spain; jlperez@unizar.es (J.L.P.-L.); joseant@unizar.es (J.A.C.); 2 GENUD (Growth, Exercise, Nutrition and Development) Research Group, 50009 Zaragoza, Spain; 3 Instituto Agroalimentario de Aragón (IA2), 50013 Zaragoza, Spain; 4 CIBEROBN Instituto de Salud Carlos III, 28029 Madrid, Spain; adipofat@gmail.com; 5 Instituto de Investigación Sanitaria Aragón, Hospital Universitario Miguel Servet, 50009 Zaragoza, Spain; jacasas@unizar.es (J.A.C.); alobosat@gmail.com (A.L.); elobo@unizar.es (E.L.); mbmoreno@unizar.es (B.M.-F.); 6 CIBERCV Instituto de Salud Carlos III, 28029 Madrid, Spain; 7 Adipocyte and Fat Biology Laboratory (AdipoFat), 50009 Zaragoza, Spain; 8 CIBERSAM Instituto de Salud Carlos III, 28029 Madrid, Spain; 9 Department of Medicine and Psychiatry, Universidad de Zaragoza, 50009 Zaragoza, Spain; 10 Department of Microbiology, Pediatrics, Radiology and Public Health, Universidad de Zaragoza, 50009 Zaragoza, Spain

**Keywords:** exercise, *APOE e4*, Cognitive Dysfunction, mild cognitive impairment

## Abstract

Physical activity (PA) reduces the risk of cognitive decline (CD) in the general population. However, little is known about whether the presence of the apolipoprotein E epsilon 4 allele (*APOE e4*) could modify this beneficial effect. The aim of this systematic review was to analyze and synthetize the scientific evidence related to PA levels and CD risk in cognitively healthy *APOE e4* carriers. Four electronic databases were analyzed. Only original articles with longitudinal study design were selected to analyze the relationship between PA and CD in *APOE e4* carriers. Five studies were included in the systematic review. All studies except one stated that PA is a protective factor against CD in *APOE e4* carriers. Moreover, partial support was found for the hypothesis that a greater amount and intensity of PA are more beneficial in CD prevention. The results support the idea that PA is a protective factor against CD in *APOE e4* carriers. Nevertheless, it would be necessary to carry out further studies that would allow these findings to be contrasted.

## 1. Introduction

Cognitive decline (CD) in older adults is an ever-growing problem because the number of older adults is increasing [1] and because longer life increases the likelihood of loss of memory and decline in the performance of cognitive tasks [2,3]. Cognitive performance is considered an important aspect of healthy aging [4]. There is an increasing interest in this subject, which is now a priority in public health [5], and the European Innovation Partnership on Active and Health Aging [6], has also recognized its relevance. Therefore, the prevention of CD should be an area of increasing interest for research in public health.

Mild cognitive impairment (MCI) is one construct of CD that has received considerable attention in the literature, mainly because it has been widely sustained that MCI progresses to dementia, Alzheimer’s disease in particular, in a high proportion of individuals [7], and also because of its high prevalence, even in the community. Several studies have suggested the idea that CD, which includes MCI, may be considered a health index because both have been associated with an increased mortality rate in the general population [8,9,10]. Moreover, it has been shown that CD is associated with disability in the general population, even after controlling for dementia [11]. Consequently, the prevention of CD should be a subject of particular attention.

A multitude of risk factors of different typology have been associated with CD [2,12,13]; age is the most important one [3], although cardiovascular diseases are also risk factors [2,12,14]. Other characteristics such as low educational level [12], depression and anxiety [10,15,16], vitamin B12 or D deficiency [12] as well as certain lifestyles that include smoking and physical inactivity [14] may also play a role in CD.

Among the previously documented risk factors of CD, a major genetic risk factor is the presence of the apolipoprotein E epsilon 4 allele (*APOE e4*) [2,17]. The human apolipoprotein E gene is polymorphic, presenting three alleles: epsilon 3 (*e3*), epsilon 2 (*e2*) and epsilon 4 (*e4*). The *e3* variant is the most frequent allele (~77% in the general population), while *e2* and *e4* are less common, with occurrence rates of 7.8% and 15.1%, respectively [18]. *APOE e4* presence is associated with several risk factors and diseases [19,20,21,22], including faster CD [2,23]. Homozygotes have the highest risk, followed by heterozygotes, implying that the effect of *APOE e4* on CD is dose-dependent [17,24]. The underlying mechanisms by which *APOE e4* is associated with early CD suggest that *APOE e4* is deficient in beta-amyloid clearance and accelerates beta-amyloid deposition to form amyloid plaques in the brain [20]. The toxic amyloid plaques injure synapses and ultimately cause neurodegeneration and CD [25].

On the other hand, protective factors of CD have also been reported [12,14,26], including intellectual activity and lifestyles aspects such as social interaction and physical activity (PA) [27,28]. PA may act on several risk factors related to CD, and a relationship between PA and neurogenesis, plasticity and higher white matter volume has been shown [29,30,31]. Several studies demonstrate how cardiovascular diseases and their risk factors are associated with CD and lower performance in multiple cognitive domains [32,33]. However, PA is beneficial in the prevention of cardiovascular diseases and their risk factors [34,35,36], as well as other known risk factors for CD such as depression or anxiety [35,37]. Therefore, PA may indirectly prevent CD by counteracting the CD risk factors. It has also been reported that PA is beneficial for the prevention of MCI and CD in the general population [12,29,31,38,39]. Recent meta-analyses also suggest that PA reduces the risk of suffering CD [27,40]; however, none of the studies reviewed were differentiated according to the genotyping of the participants based on the presence of *APOE e4*. This is a source of concern, since perhaps the effect of PA in subjects with high genetic risk of CD, *APOE e4* carriers, is different from the effect that PA has in subjects without genetic risk, *APOE e4* non-carriers. In order to create useful strategies for the prevention of severe neurodegenerative diseases, it is crucial to investigate the role that PA may have against neurodegeneration in these subjects with increased genetic risk.

Some studies have analyzed to what extent the amount and intensity of PA influence its potential beneficial effect for the prevention of CD in the general population. Several studies have suggested that higher levels of PA (exercise engaged in ≥3 days/week at intensity greater than walking) or vigorous intensity PA is more beneficial [41,42,43,44], but discrepant results have also been reported [40], and the question is not settled.

On the basis of the previous reports, the main aim of this systematic review was to analyze and synthetize the scientific evidence related to PA levels and CD risk in cognitively healthy *APOE e4* subjects (without previous CD) and to review the literature that investigates whether the effect of PA in these subjects is directly associated with amount and intensity.

## 2. Materials and Methods

This work used the model of preferred reporting items for a systematic review and meta-analysis (PRISMA) [45] to ensure accuracy and comprehensiveness. A review protocol was written prior to reviewing the literature.

As all analyses were based on publicly available summary statistics, no ethical approval from an institutional review board or informed patient consent was required. 

Four electronic databases were analyzed systematically using different keywords and Boolean operators. The databases analyzed were PubMed, SportDiscus, Cochrane Library and Web of Science (WOS). The strategy included searching by index terms (MeSH) and by free text. The search by index terms used in PubMed was: “Exercise” [Mesh] AND “Cognitive Dysfunction” [Mesh] AND “Apolipoprotein E4” [Mesh]. The search by free text used in PubMed, SportDiscus, Cochrane Library and WOS was: (“Physical Activity” OR “Exercise”) AND (“Memory impairment” OR “Age-associated memory impairment” OR “Late-life forgetfulness” OR “age-related cognitive decline” OR “Mild Cognitive Impairment” OR “Cognitive Decline” OR “Cognitive Dysfunction”) AND (“Apolipoprotein E4” OR “Apoe4” OR “Apo E4” OR “Apoe 4” OR “Apoe epsilon 4” OR “Apolipoprotein E-4” OR “Apo E-4” OR “Apo E 4” OR “Apolipoprotein E epsilon4”).

For the present systematic review, only original articles published up to 13 April 2021 were analyzed, excluding all types of symposium reports, letters to the editor, conference abstracts, books, opinions of experts and reviews of any kind. The inclusion criteria used were that the studies covered the theme of the present review (relationship between the risk of CD (which includes cases of MCI), and PA in participants genotyped with *e4* allele), were longitudinal studies, used humans as participants, were published in English and that the sample used was composed of people without brain injuries or diagnosed mental disorders at baseline.

Studies were excluded if they did not provide information on how the cognitive function or the PA was evaluated, if the PA was evaluated jointly with the rest of the activities carried out in the free time (including sedentary leisure activities) or if they were related to dementia and not specifically to CD. Papers that did not specifically assess the impact of PA on CD in *APOE e4* and those related to animal experimentation were all excluded.

**Figure 1:**
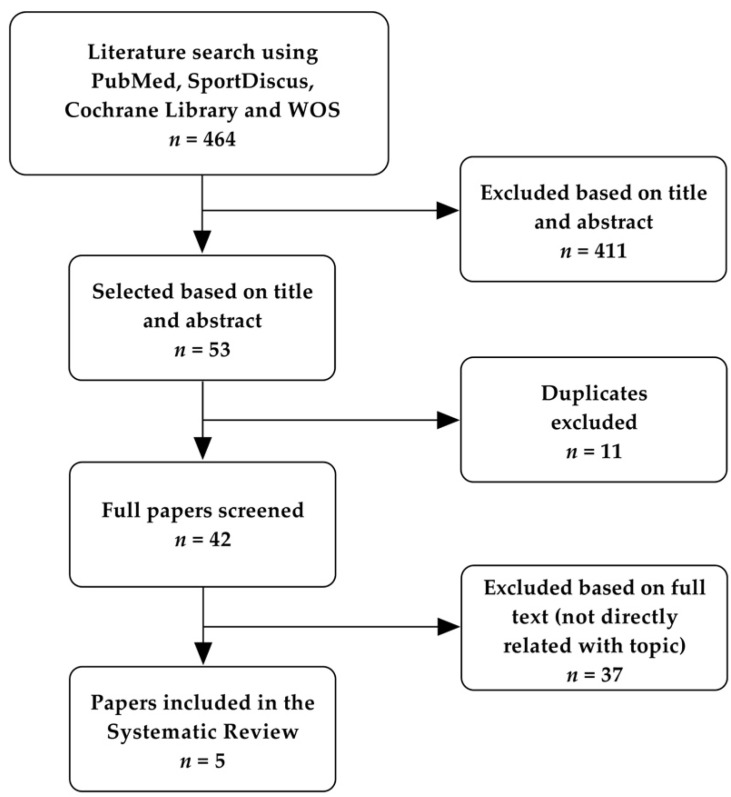
Flow diagram of search strategy in databases (PubMed, SportDiscus, Cochrane Library and Web of Science). Note: WOS—Web of Science.

Applying these criteria and using the search strategy described, the process of article selection is illustrated in Figure 1.

Two reviewers (J.L.P.-L. and A.G.A.) carried out the selection process independently based on the criteria previously established. Any discrepancies were resolved by consensus, and in some cases, a third reviewer (J.A.C.) was consulted to resolve disagreements. Starting from the initial search after applying the search criteria and strategies, the articles were first selected based on the title and abstract, identifying key and thematic keywords related or unrelated to our aim, then those duplicated articles were eliminated, and the last screening was performed after a complete and exhaustive reading of the full text, selecting the papers to be included in our systematic review. Finally, after the article selection process, the bibliography of the articles was reviewed in order to identify articles that could meet our inclusion criteria.

Data from the studies, such as the longitudinal follow-up time, the population sample used and its characteristics (average age of the participants in the baseline, sex and genotype), the cognitive function and PA evaluation method, the covariates (confounders) used in the studies, the study design and the main results, were extracted based on a second reading of the full text of the articles included in the systematic review. Moreover, the results of the included studies were grouped according to whether they compared the risk for CD among different PA levels only in *e4* carriers or in *e4* carriers vs. non-carriers. Participants of the studies were considered *APOE e4* carriers if they carried at least one *e4* allele. 

Quality assessment was performed using the Newcastle–Ottawa Scale (NOS) for cohort studies [46] and the PEDro scale for randomized controlled trials [47]. The NOS for cohort studies uses a “star” rating system to judge quality based on three aspects of the study: (a) selection of participants, (b) comparability of study groups and (c) outcome of interest. The maximum number of stars a study may receive in each of these three categories is 4, 2 and 3, respectively. The highest rating a study can receive is 9 stars. A detailed description of the NOS criteria for assessing quality can be found in the Supplementary Materials.

The PEDro scale consists of 11 criteria that assess the methodological quality of the experimental studies based on three aspects: internal validity (criteria 2–9), interpretability (criteria 10–11) and external validity or applicability (criterion 1). 

In the NOS, we assigned scores of 0–3, 4–6 and 7–9 to indicate low, moderate and high-quality studies, respectively, and, in the PEDro scale, we assigned scores of 0–4, 5–8, and 9–11 to indicate low, moderate and high-quality studies, respectively.

## 3. Results

### 3.1. Study Selection

As shown in Figure 1, the initial electronic database search yielded 464 hits in total. A total of 411 articles were excluded following screening titles and abstracts, 11 articles were removed for duplicates and 37 articles were excluded following screening of full texts. A total of five articles from the electronic search met the inclusion criteria, and, after the manual search, no articles were added.

### 3.2. Description of Included Studies

There are several differences in the design and the methodology of the different included studies. Four of them have a prospective cohort design [48,49,50,51], and the other is a randomized controlled trial [52]. Between-study differences were observed in the methods to assess the exposure and outcome. Moreover, the range of follow-up time among the studies is quite broad, from 18 months [51] to 10 years [52].

Several studies were carried out on Americans [48,50,52], one on Mexican-Americans [50] and another on American populations of Indian, African, Asian and Hispanic descent [52]. In the latter study, it should be noted that all the participants in the baseline had type II diabetes and overweight or obesity [52]. Another study was conducted on Caucasians [51], and the last one was carried out on Asians [49].

Regarding all the participants of the studies that compared the risk for CD among different PA levels in *e4* carriers vs. non-carriers (Table 1), a total of 7148 participants were included, and 1425 of them were *APOE e4* carriers, 5043 were non-carriers and the remaining 680 were reported as having a missing or unknown genotype (nine study participants from one article [48], and 671 study participants from another [52]). Moreover, the population sample of the studies ranged from 78 participants [51] to 3802 participants [52].

Taking into account all the participants of the studies that compared the risk for CD among different PA levels in *e4* carriers (Table 2), a total of 792 participants were included, but the samples of the studies also varied between 26 participants [51] and 474 participants [48].

### 3.3. Assessment of Main Variables 

To assess PA, different types of questionnaires were used. Two of the studies used questionnaires created ad hoc that were not validated [49,50], while the others used validated questionnaires such as the Minnesota Leisure Time Physical Activity Questionnaire and the 1985 National Health Interview Survey [48], Paffenbarger Physical Activity Questionnaire [52] or the Stanford Brief Activity Survey [51].

To assess cognitive function, all the studies used specific tests or questionnaires, and although there is considerable diversity, in all the studies except one [48], the Mini-Mental State Examination (MMSE) [49,51], or its modified version, the Modified Mini-Mental State Examination (3MSE) [50,52], were used, both of which are employed worldwide [53].

Table 1 and Table 2 show the questionnaire used in each of the studies to assess PA, cognitive function and the criteria for establishing CD or MCI based on the cognitive assessment tests used throughout the follow-up. Three of the included studies used MMSE or 3MSE cutoff points to define CD [49,50,52], another used Petersen’s criteria for MCI [48] and the remaining study used cutoff points in at least one of the principal outcome indices (DRS-2 and RAVLT) [51]. Table 1 and Table 2 display the different criteria used in each study to classify the participants among the different PA levels.

### 3.4. Risk of Bias Assessment

There was a heterogeneous quality in the methodology of the included studies, since two had high quality [48,50], and the others had moderate quality [49,51,52] (Table 3).

### 3.5. Risk of CD among Different PA Levels

Two tables summarize the results of the included studies. Table 1 compares the studies that analyze the risk in *APOE e4* carriers vs. non-carriers in relation to PA levels, and Table 2 compares the studies that analyze the risk between carriers with different PA levels.

Regarding the results provided in Table 1, Shih et al. stated that those carrying *APOE e4* who accomplished more than 35 MET-hours/week had a 2.20-fold increased hazard ratio (HR) (95%CI: 1.29, 3.74, *p* < 0.05), and those carrying *APOE e4* who performed less than 35 MET-hours/week had a 3.44-fold increased HR (95%CI: 1.85, 6.39, *p* < 0.05) of developing cognitive impairment compared to persons who were not *APOE e4* carriers and who performed more than 35 MET-hours/week [50]. Krell-Roesch et al. stratified their results based on the intensity of PA (light, moderate and vigorous) and also based on the moment in life when PA was performed (midlife or late life). Based on this stratification, no differences in MCI risk were found for light and vigorous PA performed in midlife or late life between *APOE e4* carriers and non-carriers (Table 1). Nevertheless, when compared to *APOE e4* non-carriers who did not perform moderate PA in midlife, *APOE e4* carriers who did not perform moderate PA in midlife had a 2.07-fold increased HR for MCI (95%CI: 1.32, 3.26, *p* < 0.05), while *APOE e4* carriers who performed moderate PA in midlife had a 1.53-fold increased HR for MCI (95%CI: 1.10, 2.15, *p* < 0.05). When compared to *APOE e4* non-carriers who did not perform moderate PA in late life, *APOE e4* carriers who did not perform moderate PA in late life had a 1.89-fold increased HR for MCI (95%CI: 1.37, 2.61, *p* < 0.05), and those *APOE e4* carriers who performed moderate PA in late life had a 1.43-fold increased HR for MCI (95%CI: 1.05, 2.95, *p* < 0.05) [48]. Woodard et al. revealed that the predicted probability of CD for *APOE e4* carriers who reported low levels of PA (≤2 days/week of low intensity) was significantly higher (*p* = 0.006) compared to *APOE e4* non-carriers who reported low or high levels of PA (≤2 days/week of low intensity, or ≥3 days/week of moderate to heavy intensity, respectively) [51]. The article by Woodard et al. also shows that the predicted probability of CD for *APOE e4* carriers who reported high levels of PA was not statistically different from that for *APOE e4* non-carriers who reported low or high levels of PA (*p* > 0.05) [51]. Espeland et al. found that *APOE e4* carriers who were involved in the PA intervention group had reduced odds for CD (OR = 0.84, 95%CI: 0.52, 1.36, *p* > 0.05) as compared with non-carriers belonging to the control group [52].

Referring to the results provided in Table 2 that compare the risk of CD among *APOE e4* carriers who showed different PA levels, Woodard et al. revealed that the predicted probability of CD for *APOE e4* carriers who reported low levels of PA (≤2 days/week of low intensity) was significantly higher (*p* = 0.006) compared to carriers who reported high levels of PA (≥3 days/week of moderate to heavy intensity) [51]. Krell-Roesch et al. stratified their results based on the intensity of the PA (light, moderate and vigorous) and also based on the moment in life when PA was performed (never, only in midlife, only in late life, or always in midlife and late life). Based on this stratification, no significant differences for suffering MCI were found among carriers who performed or who did not perform light or moderate PA in different moments of their lifespan; however, those carriers who had performed vigorous intensity activity in midlife and late life (always) had lower risk of developing MCI in comparison with those who never performed vigorous PA: HR = 0.46 (95%CI: 0.22, 0.95, *p* < 0.05) [48]. Niti et al. reported that participants who performed at least one PA had 0.34 odds for CD (95%CI: 0.17, 0.68, *p* < 0.05) compared with participants who did not perform any PA [49].

## 4. Discussion

In relation to the aims of this systematic review, the results support the notion that PA is a protective factor against CD in *APOE e4* carriers independently of the methodology used to assess the PA or the criteria to establish CD and PA levels, since this is confirmed by four of the five studies included in this systematic review. It is remarkable in this first review of previous reports in the literature that PA was effective in preventing CD in high-risk individuals such as *APOE e4* carriers. However, there are several issues related to the methodology of included studies that must be taken into account in the interpretation of the results.

As can be seen in Table 1 and Table 2, all the studies except one [51] showed results adjusted by different confounders, the most common being age and educational level. However, only two studies carried out a follow-up period >5 years [50,52]. Moreover, the short period of follow-up in some cases, the >20% loss of participants, the self-reported PA and the fact that the results were not adjusted by important confounders such as age, educational level or cardiovascular risk factors could affect the results.

The methodology used to assess PA in the studies had some weaknesses, as the researchers used questionnaires, which are based on self-reported PA and, therefore, may over- or underestimate participants’ PA. Some advantages of this type of questionnaire in studies such as those included in the present review are apparent, such as their simplicity, low cost, and ease of administration in large samples in a short period of time [54]. There is wide recognition that the choice of method may be a trade-off between accuracy level and feasibility [55], but when the aim is establishing a dose–response relationship, the use of motion sensors such as accelerometers would be important, although their use in large population studies is less feasible and they are not 100% accurate [54,56,57].

In addition, as can be seen in Table 1 and Table 2, the use of different questionnaires to assess PA among studies, and the use of different cutoff points to define the different PA levels, make it difficult to compare among studies. Moreover, the heterogeneity in the method to assess cognitive function and in the definition and criteria used for establishing CD or MCI also complicates the comparison among studies. Nevertheless, the comparability of studies is supported to some extent because these criteria are commonly used in clinical practice to confirm the presence of CD.

Furthermore, most of the included studies only specified if the participants were *APOE e4* carriers, and therefore cases, or were *APOE e4* non-carriers, and therefore controls, but did not specify whether there were any *APOE e2* carriers in control groups. This could be of interest, because, as evidence suggests, *APOE e2* has been associated with a reduced CD risk [58].

Despite these circumstances, all the included studies except one [52] support the idea that PA is a beneficial factor in terms of CD. The absence of significant differences in this study might be due to different reasons: first, the different type of sample used compared to the rest of the included studies, since all the participants had type 2 diabetes and overweight or obesity, and these are independent risk factors for CD [59] and can be improved with PA. Second, the intervention in this study may not be long enough to observe differences. Third, the results were only adjusted by age.

### 4.1. Physical Activity Dose and Risk of CD

Regarding the results of the studies, this review found that some studies suggest that an increased amount and/or intensity of PA is more effective in reducing the risk of CD for *APOE e4* carriers [48,50,51]. This finding may be explained with previous reports, which suggest that higher levels of PA may be associated with mitigating the increased risk of beta-amyloid deposition in *APOE e4* carriers [60].

However, the sample size in studies such as the one by Woodard et al. was small [51], and PA amount and intensity were only self-reported, such that the precision of the dose–response relationships may be affected. Therefore, although this review finds some support for the hypothesis that an increased amount and intensity of PA is more protective against CD in *APOE e4* carriers, the evidence is limited, and new research may be needed to document the precise amount and intensity of PA to recommend in *APOE e4* carriers.

It could be conceivable that previous studies in the general population, non-stratified by *APOE e4* status, might shed some light on this subject. According to several longitudinal studies, exercise intensity might be more beneficial than duration regarding cognitive function in the general population [41,42]. However, analyzing some studies that reported results related to the amount and intensity of PA adequate for the prevention of CD in the general population, contradictory results were found. While some studies suggest that moderate intensity seems to be sufficient to show a beneficial effect, although higher intensity is more effective [27,61], others stated that light intensity, such as a leisurely walk after dinner, is better than vigorous PA for the prevention of MCI [48].

It could be thought that the optimal dose of PA for *APOE e4* persons to prevent CD should not be very different from that of *APOE e4* non-carriers. However, according to Shih et al., the same PA level in *APOE e4* carriers and non-carriers results in a different risk reduction in CD, obtaining greater benefits for non-carriers [50]. Therefore, it is possible that carriers should perform more PA to reduce the risk to the same extent as non-carriers, but, on the other hand, a study conducted by Schuit et al. reported that, when performing the same PA level in both groups, *APOE e4* carriers obtain greater risk reduction for CD than non-carriers [62]. It is, therefore, apparent that new studies are required to determine the appropriate dose of PA to recommend in preventing CD.

### 4.2. Strengths and Limitations

The major strength of this review, which used the PRISMA system, is that, to our knowledge, it is the first systematic review focused on studies that stratify by apolipoprotein E genotype in relation to the association of PA and CD. Despite this, there are other limitations that must be considered. First, there is a heterogeneous methodology among the studies to assess the main variables and define the outcome. In the five included studies, a total of six different questionnaires were used to assess the PA, and a total of five different criteria were used to classify the participants in terms of PA levels; further, different criteria to diagnose CD were used in the included studies. Second, as discussed above, there are no studies that assess the PA with an objective method. Third, there are few studies in the literature related to our topic that show results by subgroups based on PA levels and the *APOE e4* status of the participants, and studies such as the one by Woodard et al. were carried out on small samples.

### 4.3. Future Recommendations

Although there is some evidence indicating that PA can be a protective factor against CD in *APOE e4* persons, future research will be needed in order to corroborate this. It seems that the literature is mainly limited by the failure to present data separately for *APOE e4* carriers and non-carriers, and more studies that stratify in groups according to the genotype and PA levels of the participants should, therefore, be conducted. It is also important to perform studies that evaluate PA objectively by means of motion sensors such as accelerometry, as, despite some limitations, it allows a more precise assessment of the amount and intensity of PA. It would also be interesting to perform intervention studies, ideally RCTs, in *APOE e4* persons in order to provide data on the amount and intensity of PA that will be optimal for the prevention/slowdown of CD in this type of population. Currently, some clinical trials such as U.S. POINTER, IGNITE and PAAD-2 [63,64,65] are focused on the role that PA and other lifestyle variables have in cognition among different types of population, including *APOE e4* carriers. The results of these clinical trials could shed some light on this area.

## 5. Conclusions

The results of the studies included in this systematic review support the idea that PA is a protective factor against CD in individuals of high genetic risk, specifically *APOE e4* carriers. These findings have high clinical and public health significance. Moreover, the results suggest that in this population, a higher dose of PA (amount and/or intensity) might have greater benefits, but it would be necessary to carry out further studies that would allow these findings to be contrasted, since the existing evidence is limited. Further studies should try to establish the optimal dose of PA to effectively and efficiently prevent CD in *APOE e4* carriers.

## Figures and Tables

**Table 1:** Summary of the reviewed articles that compare the risk for CD among different PA levels in *APOE e4* carriers vs. non-carriers.

Study, (Study Design)	Follow-Up, y, Mean, (Range)	Method to Assess PA	Method to Assess Cognitive Function	Adjudication of CD or MCI	Confounders	Study Subgroups	Main Results		Sample				
									*n*	Female Sex, *n* (%)	Age, y, mean (SD), and/or Range	*APOE e4*, *n*, (*e4e4*, *n*)	No *APOE e4*, *n*
Espeland, M. et al. (intervention)	9.8 (8.4–11.1)	- Paffebarger Physical Activity Questionnaire	- 3MSE - FAQ	3MSE test score fell below prespecified age- and education-specific cutoff points	- Age	- Groups by: - Genotype - Randomization: - Intervention: >175 min PA/week brisk walking - Control: PA, diet and social support	- Non-carriers control group OR = 1.00 (ref. group) - Carriers control group OR = NI - Carriers intervention group OR = 0.84 (95%CI: 0.52, 1.36, *p* > 0.05)		3802	2323 (61)	45–76	724 (57)	2407
Krell, J. et al. (observational)	3.2 (1.9–4.7)	- Minnesota leisure time physical activity questionnaire - 1985 National Health Interview Survey	- Neurological evaluation - STMS - WAIS-R - WMS-R	Petersen criteria for MCI 2004	- Age - Sex - Educational level - Comorbidities - Depression	- Groups by: - Genotype - Intensity of PA: - LPA - MPA - VPA - Moment in life when PA was performed: - Midlife - Late life	Midlife and LPA	- Non-carriers and not LPA: HR = 1.00 (ref. group) - Carriers and not LPA: HR = 1.32 (95%CI: 0.70, 2.50, *p* > 0.05) - Carriers and LPA: HR = 0.97 (95%CI: 0.66, 1.43, *p* > 0.05)	1830	919 (50.2)	78 74–83	474 (NI)	1347
							Midlife and MPA	- Non-carriers and not MPA: HR = 1.00 (ref. group) - Carriers and not MPA: HR = 2.07 (95%CI: 1.32, 3.26, *p* < 0.05) - Carriers and MPA: HR = 1.53 (95%CI: 1.10, 2.15, *p* < 0.05)					
							Midlife and VPA	- Non-carriers and not VPA: HR = 1.00 (ref. group) - Carriers and not VPA: HR = 1.84 (95%CI: 1.42, 2.39, *p* < 0.05) - Carriers and VPA: HR = 1.32 (95%CI: 0.93, 1.89, *p* > 0.05)					
							Late life and LPA	- Non-carriers and not LPA: HR = 1.00 (ref. group) - Carriers and not LPA: HR = 2.03 (95%CI: 1.23, 3.35, *p* < 0.05) - Carriers and LPA: HR = 1.35 (95%CI: 0.95, 1.91, *p* > 0.05)					
							Late life and MPA	- Non-carriers and not MPA: HR = 1.00 (ref. group) - Carriers and not MPA: HR = 1.89 (95%CI: 1.37, 2.61, *p* < 0.05) - Carriers and MPA: HR = 1.43 (95%CI: 1.05, 2.95, *p* < 0.05)					
							Late life and VPA	- Non-carriers and not VPA: HR = 1.00 (ref. group) - Carriers and not VPA: HR = 1.90 (95%CI: 1.51, 2.40, *p* < 0.05) - Carriers and VPA: HR = 1.18 (95%CI: 0.67, 2.07, *p* > 0.05)					
Shih, I. et al. (observational)	6.5	- MET-h/week of 18 common activities for older adults (based on the Compendium of Physical activities) (not validated)	- 3MSE - SEVLT (Delayed word recall) - SENAS	Score 3MSE or SEVLT fell less than the 20th percentile/decreased ≥8 in 3MSE or ≥3 points in SEVLT and scores less than 20th percentile at follow-up	- Age - Sex - Educational level - Diabetes - Smoking - History of stroke - Hours standing/walking at work	Groups by: - Genotype - Level of PA: - Low PA: <35MET-h/week - High PA: >35MET-h/week	- No *APOE e4* and High PA: HR = 1.00 (ref. group) - No *APOE e4* and Low PA: HR = 1.39 (95%CI: 0.94, 2.07, *p* > 0.05) - *APOE e4* and High PA: HR = 2.20 (95%CI: 1.29, 3.74, *p* < 0.05) - *APOE e4* and Low PA: HR = 3.44 (95%CI: 1.85, 6.39, *p* < 0.05)		1438	840 (58)	69.7 (6.2)	201 (11)	1237
Woodard, J.L. et al. (observational)	1.5	- Stanford Brief Activity Survey	- MMSE - GDS - MDRS-2 - RAVLT	≥1 SD reduction on at least one of the principal outcomes indices (DRS-2, RAVLT Sum of trials 1–5, RAVLT delayed word recall)	NI	Groups by: - Genotype - Level of PA: - Low: ≤2 d/week of low intensity (does not meet ACSM recom.) - High: ≥3 d/week of moderate to heavy intensity (meets ACSM recom.)	- *APOE e4* Low-PA demonstrated higher probability of decline than No-*APOE e4* Low-PA; *APOE e4* High-PA and No-*APOE e4* High-PA (all *p* < 0.05) - *APOE e4* High-PA probability of CD was not statistically different compared to No-*APOE e4* Low-PA and No-*APOE e4* High-PA (*p* > 0.05)		78	57 (73)	72.6 (5.0)	26 (1)	52

Abbreviations: n = sample size; y = years; NI = Not Informed; STMS = Short Test of Mental Status; WAIS-R = Wechsler Adult Intelligence Scale—Revised; WMS-R = Wechsler Memory Scale—Revised; PA = Physical Activity; LPA = Light Physical Activity; MPA = Moderate Physical Activity; VPA = Vigorous Physical Activity; HR = Hazard Ratio; MET = Metabolic Equivalent; h = hours; d = days; min = minutes; 3MSE = Modified Mini Mental Status Examination; SEVLT = Spanish English Verbal Learning Test; SENAS = Spanish English Neuropsychological Assessment Scale; FAQ = Functional Assessment Questionnaire; OR = Odds Ratio; MMSE = Mini Mental State Examination; GDS = Geriatric Depression Scale; MDRS-2 = Mattis Dementia Rating Scale-2; RAVLT = Rey Auditory Verbal Learning Test; SD = Standard Deviation; ACSM = American College of Sports Medicine; recom. = recommendations.

**Table 2:** Summary of the reviewed articles that compare the risk for CD among different PA levels in *APOE e4* carriers.

Study, (Study Design)	Follow-Up, y, Mean, (Range)	Method to Assess PA	Method to Assess Cognitive Function	Adjudication of CD or MCI	Confounders	Study Subgroups	Main Results		Sample		
									*n*, (*e4e4*, *n*)	Female Sex, *n* (%)	Age, y, Mean (SD), and/or Range
Krell, J. et al. (observational)	3.2 (1.9–4.7)	- Minnesota leisure time physical activity questionnaire - 1985 National Health Interview Survey	- Neurological evaluation - STMS - WAIS-R - WMS-R	Petersen criteria for MCI 2004	- Age - Sex - Educational level - Comorbidities - Depression	Groups by: - Intensity of PA: - LPA - MPA - VPA - Moment in life when PA was performed: - Never - In midlife - In late life - Always	LPA	- Never LPA: HR = 1.00 (ref. group) - Only LPA in midlife: HR = 0.80 (95%CI: 0.33, 1.98, *p* > 0.05) - Only LPA in late life: HR = 0.59 (95%CI: 0.19, 1.82, *p* > 0.05) - Always LPA: HR = 0.56 (95%CI: 0.26, 1.21, *p* > 0.05)	474 (NI)	NI	NI
							MPA	- Never MPA: HR = 1.00 (ref. group) - Only MPA in midlife: HR = 0.90 (95%CI: 0.52, 1.55, *p* > 0.05) - Only MPA in late life: HR = 0.96 (95%CI: 0.43, 2.13, *p* > 0.05) - Always MPA: HR = 0.68 (95%CI: 0.40, 1.14, *p* > 0.05)			
							VPA	- Never VPA: HR = 1.00 (ref. group) - Only VPA in midlife: HR = 0.87 (95%CI: 0.57, 1.33, *p* > 0.05) - Only VPA in late life: HR = 1.00 (95%CI: 0.40, 2.49, *p* > 0.05) - -Always VPA: HR = 0.46 (95%CI: 0.22, 0.95, *p* < 0.05)			
Niti, M. et al. (observational)	(1–2)	- Frequency of participation in physical exercise routines; walking; active sports; tai chi (not validated)	- MMSE	Decline ≥1 points in MMSE between baseline and follow-up	- Age - Sex - Educational level - Number of comorbidities - Functional status - Vascular risk factors - Depression - Smoking - Alcohol	Groups by: - At least one PA: - Yes - No	- No-PA OR = 1.00 (ref. group) - Yes-PA OR = 0.34 (95%CI: 0.17, 0.68, *p* < 0.05)		292 (NI)	NI	NI
Woodard, J.L. et al. (observational)	1.5	- Stanford Brief Activity Survey	- MMSE - GDS - MDRS-2 - RAVLT	≥1 SD reduction on at least one of the principal outcomes indices (DRS-2, RAVLT Sum of trials 1–5, RAVLT delayed word recall)	NI	Groups by: - Level of PA: - Low: ≤2 d/week of low intensity (does not meet ACSM recom.) - High: ≥3 d/week of moderate to heavy intensity (meets ACSM recom.)	- Low-PA group demonstrated higher probability of decline than High-PA group (*p* < 0.05)		26 (1)	NI	NI

Abbreviations: n = sample size; y = years; STMS = Short Test of Mental Status; WAIS-R = Wechsler Adult Intelligence Scale; WMS-R = Wechsler Memory Scale; PA = Physical Activity; LPA = Light Physical Activity; MPA = Moderate Physical Activity; VPA = Vigorous Physical Activity; HR = Hazard Ratio; MET = Metabolic Equivalent; h = hours; d = days; min = minutes; 3MSE = Modified Mini Mental Status Examination; SEVLT = Spanish English Verbal Learning Test; FAQ = Functional Assessment Questionnaire; OR = Odds Ratio; MMSE = Mini Mental State Examination; GDS = Geriatric Depression Scale; MDRS-2 = Mattis Dementia Rating Scale-2; RAVLT = Rey Auditory Verbal Learning Test; ACSM = American College of Sports Medicine; recom. = recommendations; NI = Not Informed.

**Table 3:** Newcastle–Ottawa and PEDro Quality Assessment of the studies included in the systematic review.

**Study**	**Quality Assessment of Cohort Studies with NOS**											
	**Selection**				**Comparability**		**Outcome**			**NOS QS**		
	**1**	**2**	**3**	**4**	**5**	**6**	**7**	**8**	**9**			
Krell, J. et al.	*	*		*	*	*	*		*	7		
Niti, M. et al.	*	*		*	*	*	*			6		
Shih, I. et al.	*	*		*	*	*	*	*	*	8		
Woodard, J.L. et al.	*	*		*			*		*	5		
**Study**	**Quality Assessment of RCT Studies with PEDro Scale**											
	**1**	**2**	**3**	**4**	**5**	**6**	**7**	**8**	**9**	**10**	**11**	**PEDro** **QS**
Espeland, M. et al.	*	*	*	*			*			*	*	7

Abbreviations: NOS = Newcastle–Ottawa Scale; QS = Quality Score; RCT = Randomized Controlled Trial; * = One point.

## Data Availability

Not applicable.

## Supplementary Materials

The following are available online at https://www.mdpi.com/article/10.3390/ijerph18147238/s1, Text S1: Quality assessment information. Newcastle–Ottawa Scale (NOS) criteria.
