# Supplementary material for: Can Physical Activity Reduce the Risk of Cognitive Decline in Apolipoprotein e4 Carriers? A Systematic Review
Source: Int J Environ Res Public Health. 2021 Jul 6;18(14):7238. doi: 10.3390/ijerph18147238 (PMC8303365; doi:10.3390/ijerph18147238)
Supplement: Supplementary file 1 [file ijerph-18-07238-s001.zip › ijerph-1211934 Supplementary.pdf]

International Journal of
*Environmental Research and Public Health*

MDPI

*Review*

# Can Physical Activity Reduce the Risk of Cognitive Decline in Apolipoprotein e4 Carriers? A Systematic Review

**Supplementary Text S1: Quality assessment information. Newcastle-Ottawa Scale (NOS) criteria**

In section (a), the representativeness of the exposed cohort, the selection of the non-exposed cohort, the ascertainment of exposure, and the demonstration that outcome of interest was not present at start of the study were evaluated and scored or not with a star.

* Representativeness of the exposed cohort:

a) truly representative of the average PA in the community →Star (1)

b) somewhat representative of the average PA in the community →Star (1)

c) selected group of users eg nurses, volunteers →No star (0)

d) no description of the derivation of the cohort →No star (0)

* Selection of the non-exposed cohort:

a) drawn from the same community as the exposed cohort →Star (1)

b) drawn from a different source →No star (0)

c) no description of the derivation of the non-exposed cohort →No star (0)

* Ascertainment of exposure:

a) secure record (eg surgical records) →Star (1)

b) structured interview →Star (1)

c) written self-report →No star (0)

d) no description →No star (0)

* Demonstration that outcome of interest was not present at start of study: 24

a) yes →Star (1)

b) no →No star (0)

In section (b), the NOS for cohort studies evaluate the variables of control that studies use to adjust their results. A star was scored for the studies that adjusted their results by the confounder age, and with another star if they also adjusted for the confounder education level or years of education received. Age was selected as the most relevant factor, supported by conclusions in several studies [1], and as second relevant factor authors selected education based on the results of a systematic review [2].

* Comparability of cohorts on the basis of the design or analysis

a) study controls for age →Star (1)

b) study controls for education level / years of education received →Star (1)

In section (c) the assessment of the outcome was taken into account, the follow-up period, and the loss of participants throughout the follow-up. The second item scored with a star in those studies in which the follow-up from the baseline was ≥5 years, and the third item scored with a star when the loss of participants in the study between the baseline and the follow-up was £20%. We believe that participant losses >20% may compromise the results of the studies, and in view of clinical experience, we also believe that 5 years can be a reasonable period of time for follow-up; moreover, other similar studies use this time-frame [3], and we think that the use of similar criteria is an advantage for comparative studies.

* Assessment of outcome:

a) independent blind assessment →Star (1)

b) record linkage →Star (1)

c) self-report →No star (0)

d) no description →No star (0)

* Was follow-up ≥5 years?:

a) yes →Star (1)

b) no →No star (0)

* Adequacy of follow-up of cohorts:

a) complete follow-up – all subjects accounted for →Star (1)

b) subjects lost £20% →Star (1)

c) follow up rate <80% and no description of those lost →No star (0)

d) no statement →No star (0)

## References

1 Lipnicki DM, Makkar SR, Crawford JD, Thalamuthu A, Kochan NA, Lima-costa MF, et al. Determinants of cognitive performance and decline in 20 diverse ethno-regional groups: A COSMIC collaboration cohort study. *PLoS Med*. **2019**, *16*, 1–27.

2 Luck T, Luppa M, Briel S, Riedel-Heller SG. Incidence of Mild Cognitive Impairment: A Systematic Review. *Dement Geriatr Cogn Disord*. **2010**, *29*, 164–175.

3 Blondell SJ, Hammersley-Mather R, Veerman JL. Does physical activity prevent cognitive decline and dementia?: A systematic review and meta-analysis of longitudinal studies. *BMC Public Health*. **2014**, *14*, 1–12.
